# Beer and its Non-Alcoholic Compounds: Role in Pancreatic Exocrine Secretion, Alcoholic Pancreatitis and Pancreatic Carcinoma

**DOI:** 10.3390/ijerph7031093

**Published:** 2010-03-15

**Authors:** Andreas Gerloff, Manfred V Singer, Peter Feick

**Affiliations:** Department of Medicine II, Universitätsmedizin Mannheim, Theodor-Kutzer-Ufer 1-3, 68167 Mannheim, Germany; E-Mails: andreas.gerloff@umm.de (A.G.); manfred.v.singer@umm.de (M.V.S.)

**Keywords:** beer, non-alcoholic constituents, pancreatitis, pancreatic carcinoma

## Abstract

In this article we provide an overview of the newest data concerning the effect of non-alcoholic constituents of alcoholic beverages, especially of beer, on pancreatic secretion, and their possible role in alcoholic pancreatitis and pancreatic carcinoma. The data indicate that non-alcoholic constituents of beer stimulate pancreatic enzyme secretion in humans and rats, at least in part, by direct action on pancreatic acinar cells. Some non-alcoholic compounds of beer, such as quercetin, resveratrol, ellagic acid or catechins, have been shown to be protective against experimentally induced pancreatitis by inhibiting pancreatic secretion, stellate cell activation or by reducing oxidative stress. Quercetin, ellagic acid and resveratrol also show anti-carcinogenic potential *in vitro* and *in vivo*. However, beer contains many more non-alcoholic ingredients. Their relevance in beer-induced functional alterations of pancreatic cells leading to pancreatitis and pancreatic cancer in humans needs to be further evaluated.

## 1. Introduction

Alcoholic beverages contain numerous non-alcoholic compounds that could have beneficial or pathological effects. For example, more than 2,000 organic and inorganic constituents in beer and more than 1,000 in wine were identified to date. Whereas the role of alcohol (ethanol) in the development of pancreatic diseases—in particular acute and chronic pancreatitis—has been intensively investigated, only little is known about the effects of non-alcoholic compounds in this context. Some of the non-alcoholic constituents have been shown to be biologically active, although the results are often not discussed in appropriate publications in regard to their consumption as an inherent mixture in alcoholic beverages.

This review summarizes the present knowledge about the effect of beer and other alcoholic beverages on the pancreas in comparison to pure ethanol. Firstly, the article focuses on investigations with beer in human pancreatic secretion *in vivo* and rat pancreatic secretion *in vitro*. The second part briefly describes the current evidence on the link between beer consumption and pancreatitis based on epidemiological data. Subsequently, data concerning the effects of selected non-alcoholic compounds of beer on experimentally-induced pancreatitis in animals are presented. Studies that deal with the cancer preventive potential of the non-alcoholic compounds of beer are discussed in the fourth part.

## 2. Results and Discussion

### 2.1. Beer and Pancreatic Secretion

Because hypersecretion of pancreatic enzymes has been observed in chronic alcoholics, a general impression is that ethanol stimulates basal (interdigestive) pancreatic exocrine secretion [1]. In the literature, various effects of alcohol consumption on pancreatic exocrine secretion have been demonstrated, which may be due to the route of administration and different experimental conditions [2]. Whereas acute oral and intragastric ethanol administration increases pancreatic bicarbonate and protein secretion, intravenous ethanol administration reduces basal and hormonally stimulated pancreatic bicarbonate and protein secretion. However, alcohol is commonly consumed as a tasty beverage that is a complex mixture with non-alcoholic compounds, a number of these which may also possibly influence pancreatic secretion.

Studies on human pancreatic secretion suggest different effects of alcoholic beverages when compared to appropriate ethanol solutions [3–5]. Intragastric administration of beer in a volume (250 mL) that does not alter plasma ethanol concentrations causes a significant stimulation of basal pancreatic enzyme output, whereas ethanol in concentrations similar to beer (4% v/v) has no effect [3]. Therefore, the non-alcoholic constituents might be responsible for the stimulatory effect of beer on pancreatic enzyme secretion in humans. The intragastric administration of an higher amount of beer (850 mL) or white wine (400 mL) elevates plasma ethanol concentrations, but does not affect the basal pancreatic enzyme output [5]. It was suggested that the direct inhibitory effect of circulating ethanol neutralized the stimulatory effect of the non-alcoholic components of beer.

In a study with healthy humans, Chari *et al.* (1996) found that isotonic glucose solution (5.76% w/v) as well as glucose solution with a concentration found in finished wort (11.5% w/v) were strong releasers of cholecystokinin (CCK), but stimulated the exocrine pancreatic secretion only moderately as compared to beer. These results suggest that CCK is one, but not the exclusive, mediator of pancreatic secretion induced by beer. However, direct effects of beer on enzyme secretion has not been examined, and can not necessarily be inferred from infusion or injection studies because of potential interaction with enteropancreatic reflexes [6], other hormones [7–9] or regulators [9,10] *in vivo*.

**Figure 1.**
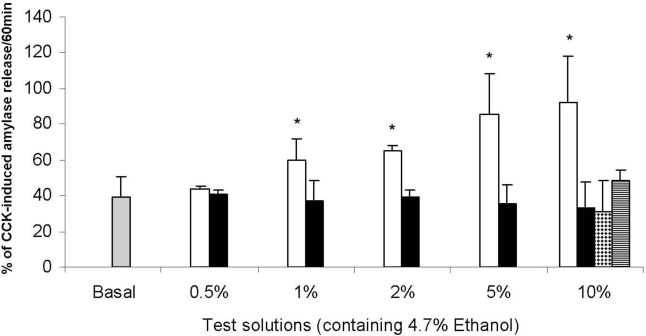
Effect of beer (white bars), ethanol (black bars), red wine (spotted bar) and whisky (striped bar) on basal amylase release in AR4-2J cells. Volumes are expressed as % of test solution in incubation buffer. Adapted from [12].

Therefore, recent studies investigated the direct effect of alcoholic beverages, especially beer, on pancreatic secretion of freshly isolated rat pancreatic acinar cells and the rat pancreatic acinar cell line AR4-2J [11,12]. The cell line retains many of the characteristics of pancreatic acinar cells in its differentiated state and is a general model to study stimulus-secretion coupling [13]. Stimulation of AR4-2J cells with different alcoholic beverages and appropriate ethanol solutions have shown that increasing beer dose increases amylase release, whereas pure ethanol, wine or alcoholic beverages produced by distillation (e.g., whisky, tequila) have no effect (Figure 1).

This effect was reproducible in freshly isolated pancreatic acinar cells. Lactate dehydrogenase measurement after long-term treatment (24 h) of AR4-2J cells indicated that the beer-induced amylase release is not due to protein release due to cell membrane damage [12]. Pre-treatment of AR4-2J cells with selective inhibitors of known mediators of stimulus-secretion coupling in pancreatic acinar cells has shown that beer-induced amylase secretion is mediated by activation of phospholipase C (PLC), binding of IP_3_ to its receptor (IP_3_-R) and subsequent calcium release from intracellular stores. The participation of calcium in beer-induced amylase release was confirmed by pre-treatment of the cells with the calcium chelator BAPTA/AM and measurement of fluorescence after loading the cells with the calcium indicator Fura-2 AM. However, further results indicate that additional, yet unknown, signaling pathways are involved in the beer-induced amylase release [11].

Concerning the characterization of the compounds that are responsible for the stimulatory effect of beer *in vitro*, we have shown that barley is the source of the stimulants and malting as well as fermentation have just a marginal effect on the stimulatory action. In addition, fermented and unfermented glucose in concentrations found in finished wort, as well as the known stimulants of gastric secretion, maleic acid and succinic acid, have no effect on pancreatic secretion of AR4-2J cells. Stimulation of cells with treated beer (distillation, lyophilization, dialysis and protease digestion) suggested that the stimulatory compound/s is/are heat-stable, non-volatile substance/s with a molecular weight higher than 15 kDa [11].

### 2.2. Beer and Pancreatitis

Alcohol is the major etiological factor of acute and chronic pancreatitis (for recent review see [14]). Alcohol is responsible for up to 40% of the acute pancreatitis cases, and 60−90% of chronic pancreatitis patients have a history of chronic alcohol consumption [15]. However, the fact that only a minority of less than 10% of heavy drinkers develop alcoholic pancreatitis [16] and that ethanol alone can not induce pancreatic inflammation in animal experiments [17] promoted an intensive search for cofactors of the development of chronic pancreatitis. These include diet, smoking, type of alcoholic beverage consumed, pattern of drinking, lipid intolerance and inherited factors [18,19].

The influence of beverage type on the development of pancreatitis remains controversial (for review see [20]). Wilson *et al.* [21] compared alcoholic pancreatitis patients with alcoholic cirrhosis patients and found a slight tendency to consume less beer and more wine in the latter. Nakamura *et al.* [22] found that spirits increased the risk for chronic pancreatitis more than sake and beer in 111 Japanese alcoholics, without significant difference in the total amount of ethanol consumed. A more recent study of the same authors reported that the amount of alcohol consumed per day was greater in spirit drinkers, suggesting that the higher alcohol consumption may be associated with the higher risk of pancreatitis [23]. The latest population-based cohort study of Kristiansen *et al.* [24] analysed a hazard ratio of 2.0 for the development of pancreatitis with drinking of more than 14 glasses of beer/week, whereas no association was observed for wine and spirits. However, this study was limited due to only a few participants with a high consumption (≥14 drinks/week) of both wine and spirits (eight and eight drinks/week, respectively) *versus* 49 drinks/week for beer.

### 2.3. Non-Alcoholic Constituents of Beer and Pancreatitis

Beer is an extremely complex beverage, and thousands of constituents have been identified hitherto. However, one of the differentiating characteristics among alcoholic beverages is their polyphenol composition. In this context, wine contains more abundant polyphenols than beer and liquors. In beer, more than 50 polyphenolic compounds were identified [25]. About 75–85% of these polyphenols derive from malt and 15–25% from hop. The polyphenol content of each beer is largely affected by the type and the sort of corn (barley, wheat), the type of hop, as well as by the different processes to produce the malt, wort and hop extract.

The best studied polyphenol is *resveratrol*, which is a phenolic phytoalexin present in grape skins and wines (especially red wines) and was also found in beer. Some recent studies demonstrated a protective effect of resveratrol on experimentally induced acute pancreatitis in animal models. Szabolcs *et al.* [26] examined the effect of resveratrol on CCK-induced acute pancreatitis in male Wistar rats. Pre-treatment with 10 mg/kg body weight resveratrol ameliorated CCK-induced changes of laboratory parameters such as amylase, lipase, glucose, calcium, creatinine and aspartate aminotransferase activities as well as the serum concentration of triglyceride and urea nitrogen. Furthermore, a reduced extent of tissue edema, acinar vacuolization and total histological damage was found by histological investigation of the pancreas after resveratrol treatment. Since no inhibition of nuclear factor (NF)-κB activation by resveratrol was found, it was concluded that the beneficial effects of resveratrol seem to be mediated by the anti-oxidant effect of resveratrol or by an anti-inflammatory mechanism independent of NF-κB.

In contrast to this, activation of NF-κB in macrophages is involved in the inflammatory response of rats with sodium taurocholate-induced severe acute pancreatitis (SAP). Resveratrol (10 mg/kg body weight) decreased the NF-κB activation as well as expression of inducible nitric oxide synthase (iNOS) in peritoneal macrophages [27]. In addition, in resveratrol-treated SAP rats, serum levels of tumor necrosis factor (TNF)-α, interleukin (IL)-1 and nitric oxide were also reduced at 3, 6 and 12 hours after induction of SAP. An attenuation of various pathological manifestations by resveratrol as compared to untreated SAP group was also found by histological examinations of the pancreas. These results were confirmed by further studies showing the inhibitory effect of resveratrol on expression of NF-κB and the levels of TNF-α and IL-8 in pancreatic tissues of SAP-rats [28], as well as reduction of intracellular calcium overload, which otherwise leads to tissue damage in this model [29].

Previous studies have shown that pancreatic damage in experimental pancreatitis can be reduced by hepatocyte growth factor (HGF). Warzecha *et al.* [30] investigated the effect of resveratrol on HGF in cerulein-induced pancreatitis in Wistar rats. In contrast to the study of Meng *et al.* [28] treatment with 10 mg/kg resveratrol alone had no effect on the development of pancreatitis. Resveratrol did not affect lipase activity, concentrations of IL-1ß and IL-10 in the plasma as well as pancreatic DNA synthesis. In addition, resveratrol did not affect the protective effect of HGF. Since it has been shown that resveratrol is an inhibitor of cyclooxygenase-1 activity, it can be concluded that this activity is not involved in the protective effect of HGF in acute pancreatitis.

Oxidative stress is another important process that may play a role in pancreatic injury. Evidence of oxidative stress has also been reported in the pancreas of patients with alcoholic chronic pancreatitis [2]. The effect of resveratrol on pancreatic oxygen free radicals was investigated in rats with severe acute pancreatitis [31]. Histological examinations showed that resveratrol treatment (20 mg/kg body weight) after induction of acute pancreatitis led to a significant reduction of turbidity ascitic fluid, pancreatic edema, necrosis and inflammatory cell infiltration as compared to the pancreatitis group. In addition, resveratrol treatment significantly diminished serum amylase, inhibited the formation of the lipid peroxidation product malondialdehyde (MDA) and increased the pancreatic superoxide dismutase (SOD, an internal anti-oxidase). Measurement of myeloperoxidase (MPO) as an indicator of neutrophil sequestration, another source of oxygen free radicals during acute pancreatitis, showed a reduction of this enzyme in the resveratrol-treated group. An attenuation of various pathological manifestations (less pronounced necrotic changes of rat pancreata such as focal edema, acinar cell vacuolization, and focal tissue necrosis) as well as a decrease of serum amylase activity were also found by treatment with 2 mg resveratrol for eight days before induction of acute pancreatitis with free radicals (tert-butyl hydroperoxide) in male Wistar rats [32].

In conclusion, resveratrol has a protective effect on pancreatic damage by lowering oxidative free radicals and reducing tissue infiltration of neutrophils. Resveratrol may be considered as an agent for reducing the inflammatory response in acute pancreatitis.

Similar effects were found for the effect of *catechins* on ethionine-induced acute pancreatitis in male Wistar rats [33]. *Catechins* are phytoalexins, which are found in green tea, various vegetables and fruits and particularly in paring of grapes of red wines as well as beer. Morphological analysis of rats that were supplied with 0.2% solution of green tea extract (GTE, 91.2% catechins) for 13 days prior to injection of 800 mg/kg ethionine, showed few lesions on pancreatic tissue except for mild interstitial edema, whereas in rats without GTE treatment inter- and intrastitial edema and necrosis of acinar cells were found. A significant decrease in serum amylase activity and tissue lipid peroxides concentration was also found in GTE-drinking rats, as compared to the ethionine-treated group supplied with water. Similar results were reported by the same author for the effects of catechins on cerulein-induced pancreatitis [34]. Certain catechins have anti-oxidative properties by eliminating superoxide and hydroxyl radicals. Thus, it could be shown that a 0.1% solution of green tea catechins as drinking water had a protective effect against the oxidative stress in pancreas and liver induced by the pancreatic carcinogen N-nitro-sobis(2-oxopropyl)amine in Syrian golden hamsters [35]. The increase of 8-hydroxydeoxyguanosine content in nuclear DNA, which is a biomarker of DNA oxidative damage, was inhibited by green tea catechins. In addition, an inhibitory effect of green tea catechins was also shown on lipid peroxidation confirming the protective effect of catechins on oxidative stress.

In contrast, the same authors showed that GTE do not suppress the lipid hydroperoxide-induced 8-oxodG (biomarker for oxidative stress and carcinogenesis) increase in pancreas, but slightly suppressed the amount in liver [36]. Taken together, catechins were shown to have a protective effect on experimental-induced pancreatitis in rats.

In cerulein-induced pancreatitis, heat shock protein 70 (HSP70) prevents secretagogue-induced cell injury in the pancreas of Wistar rats by preventing intracellular trypsinogen activation [37]. Incubation of cultured pancreas fragments with 50 μM *quercetin* completely blocked culture-induced upregulation of HSP70 expression and restored the culture-dependent loss of trypsinogen activation in the cerulein-induced pancreatitis. *Quercetin* is a naturally occurring flavonoid present in a variety of plants and in beer and wine and has important anti-degenerative properties [38]. Studying non-pancreatic tissues it was shown that quercetin inhibits synthesis of HSP110, HSp90, HSP40, and HSP28, in addition to HSP70.

Pancreatic stellate cells (PSCs) are the main source of extracellular matrix synthesis leading to pancreatic fibrosis and are activated by growth factors, inflammatory cytokines, alcohol, its metabolite acetaldehyde and oxidative stress [39]. Recently, it has been shown, that *ellagic acid* has crucial effects on a number of cell functions including activation of PSCs *in vitro* [40]. *Ellagic acid* is a polyphenol mainly found in fruits (raspberries, strawberries), nuts (walnuts) and wood, but also in beer and in wine [25,40]. Ellagic acid inhibited the platelet derived growth factor (PDGF)-induced proliferation and migration in a dose-dependent manner (1−25 μg/mL) without affecting cell viability. At a concentration of 10 μg/mL, ellagic acid inhibited PDGF-induced tyrosine phosphorylation of PDGF ß-receptor and the activation of the downstream signalling pathways (extracellular signal-regulated protein kinase and Akt). Furthermore, ellagic acid significantly inhibited several key functions of PSCs including activator protein-1, α-smooth muscle actin gene expression, monocyte chemoattractant protein-1 production and collagen expression in a dose-dependent manner. In addition, the transformation of freshly isolated PSCs from quiescent to myofibroblast-like phenotyp in culture was also blocked by ellagic acid. Because of these crucial effects on cell functions and the activation of PSCs ellagic acid is a potential candidate for the treatment of pancreatic fibrosis and inflammation.

### 2.4. Beer and Carcinogenesis

Pancreatic carcinoma, particularly pancreatic ductal carcinoma, is characterized by extremely aggressive behavior, with an overall five-year survival of less than 4% [41]. Since therapeutic specimens have only little impact on patient survival, epidemiological studies and molecular research focus on identification and reduction of risk factors. Epidemiological data suggest a slight, but not significant increase in risk for pancreatic carcinoma for individuals consuming 30 or more grams alcohol per day [42]. However, there is an indirect relationship, since ethanol induces the development of chronic pancreatitis and this can lead to pancreatic carcinoma [43]. The cumulative risk for the development of pancreatic carcinoma in the presence of chronic pancreatitis for 10 and 20 years is equal to 1.8% and 4.0%, respectively. The risk is not related to the sex of the patients, the geographical location, or the etiology of pancreatitis [43]. Therefore, chronic pancreatitis can be regarded as a precancerous disease.

Flavonols are a class of flavonoids, polyphenols, which are ubiquitous in plant foods and are known compounds of beer. A recent cohort study evaluated the participants’ food consumption and calculated the intake of the three flavonols: quercetin, kaempferol, and myricetin. These results showed that flavonol intake reduces the risk for developing pancreatic cancer [44].

In addition, different polyphenols (quercetin, ellagic acid and resveratrol) were shown to have an inhibitory effect on pancreatic cancer growth *in vivo* and *in vitro* (Table 1). In detail, treatment with a daily dose of quercetin (1.3 mg i.p.) in a nude mouse model of pancreatic cancer as well as incubation of pancreatic carcinoma cell lines (human Mia PACA-2, rat BSp73AS) with 100 μM quercetin, showed multiple effects on pancreatic cancer: prevention of metastatic lesions, decrease of primary tumor growth and induction of apoptosis in tumor cells [45].

Ellagic acid at concentrations of 10−50 mmol/L stimulated apoptosis in human pancreatic Mia PACA-2 and PANC-1 tumor cells. In addition, ellagic acid decreased proliferation by up to 20-fold at 50 mmol/L and stimulated the mitochondrial pathway of apoptosis associated with mitochondrial depolarization, cytochrome C release, and the downstream caspase activation. In a dose-dependent manner, ellagic acid decreased NF-kB binding activity; inhibition of NF-kB activity using IkB prevented the effect of ellagic acid on apoptosis. Therefore, the data indicate that ellagic acid stimulates apoptosis through inhibition of the pro-survival transcription factor NF-kB [46].

Further experiments indicated that also resveratrol but not rutin (glycosidic form of quercetin and one of the flavonoids most abundant in foods) activate apoptosis in pancreatic cancer cells [45]. The results showed an inhibitory effect of resveratrol on pancreatic cancer growth by inducing mitochondrial dysfunction followed by cytochrome c release, caspase activation and apoptosis. In addition, resveratrol sensitized the cells to ionizing radiation [49]. A potent anti-proliferative effect of 100 μM resveratrol has also been described in human pancreatic cancer cell lines PANC-1 and AsPC-1 [47].

A protective effect has also been found for proanthocyanidins on 2-amino-1-methyl-6-phenylimidazo[4,5-b]pyridine (PhIP)-induced mutagenesis *in vitro* and on *in vivo* carcinogenesis in Sprague-Dawley rats [48]. *Proanthocyanidins* can be found in many plants, most notably apples, pine bark, cinnamon, grape seed, cocoa, grape skin, red wines and beer. The eight-fold treatment with PhIP (100mg/kg body weight) over a period of four weeks resulted in acinar lesions in exocrine pancreas of all treated rats, which decreased dose-dependently by proanthocyanidins given during the initiation period. Colon lesions were not observed and pituitary lesions such as hyperplasia, adenoma and hematoma, liver cell foci, uterine stromal polyps, small intestinal adenocarcinomas, and leukemia were sporadically found in the PhIP-treated rats, but proanthocyanidins did not appear to affect their development.

## 3. Conclusion

Beer, but not other alcoholic beverages or ethanol, stimulates pancreatic secretion in humans. This effect is, at least in part, mediated by direct effects of non-alcoholic compounds of beer on pancreatic acinar cells. It is conceivable that beer-induced alterations of the stimulus-secretion coupling contribute to the doubled risk to develop chronic pancreatitis for individuals with a daily high beer intake, which is described by Kristiansen *et al.* [24].

Single compounds of beer show rather a beneficial effect on experimentally-induced pancreatitis and pancreatic carcinoma. However, beer is an extremely complex mixture of bioactive substances and just a fraction of these compounds were tested for their effect/s on pancreatitis or pancreatic carcinoma. In addition, future studies should also focus on defined combinations of substances in relevant concentrations, because one may assume that the effect/s of the mixture will differ from the effect of single components.

## Figures and Tables

**Table 1.** Studies on the effects of non-alcoholic constituents of beer on pancreatic carcinoma.

**Compound**	**Experimental Design**	**Effect on Pancreas**	**Species**	**Reference**
**Quercetin**	*in vivo* 1.3 mg daily, initiated 10 days after tumor transplantation	Decrease of the primary tumor growth Prevention of metastatic lesion	Nude Mouse	[45]
	*in vitro* <100 μM	Induction of apoptosis by mitochondrial depolarization, cytochrome c release and caspase activation	Rat (BSp73AS cell line) Human (Mia PACA-2 cell line)	[45]
**Ellagic acid**	*in vitro* 10–50 mM	Induction of apoptosis by mitochondrial depolarization, cytochrome c release and caspase activation Decrease of NF-kB binding activity	Human (Mia PACA-2 and PANC-1 cell line)	[46]
**Resveratrol**	*in vitro* <100 μM	Induction of apoptosis by mitochondrial depolarization, cytochrome c release and caspase activation	Rat (BSp73AS cell line) Human (Mia PACA-2 cell line)	[45]
	*in vitro* 100 μM	Inhibition of proliferation and induction of apoptosis Change of cell cycle distribution	Human (PANC-1 and AsPC-1 cell line)	[47]
**Proanthocyanidins**	*in vivo* 0.025 and 0.25% fed during or after experimentally induced tumor growth	Inhibition of rat pancreatic carcinogenesis in the initiation stage	Rat (Sprague-Dawley)	[48]
